# Small Functional Foods: Comparative Phytochemical and Nutritional Analyses of Five Microgreens of the Brassicaceae Family

**DOI:** 10.3390/foods10020427

**Published:** 2021-02-15

**Authors:** Ilaria Marchioni, Marco Martinelli, Roberta Ascrizzi, Costanza Gabbrielli, Guido Flamini, Luisa Pistelli, Laura Pistelli

**Affiliations:** 1 Department of Agriculture, Food and Environment, University of Pisa, via del Borghetto 80, 56126 Pisa, Italy; ilaria.marchioni.16@gmail.com (I.M.); laura.pistelli@unipi.it (L.P.); 2 PlantLab, Institute of Life Sciences, Scuola Superiore Sant’anna, via Guidiccioni 8-10, 56010 San Giuliano Terme, Italy; marco.martinelli@santannapisa.it; 3 Pharmacy Department, University of Pisa, via Bonanno 6, 56026 Pisa, Italy; costi.g89@hotmail.it (C.G.); guido.flamini@unipi.it (G.F.); luisa.pistelli@unipi.it (L.P.); 4 Interdepartmental Research Center “Nutraceuticals and Food for Health”, University of Pisa, via del Borghetto 80, 56126 Pisa, Italy

**Keywords:** essential oil, isothiocyanates, ascorbic acid, polyphenols, anthocyanins, antioxidant activity, carotenoids, rocket salad, broccoli, daikon, mustard, watercress, arugula, white radish

## Abstract

Microgreens are the seedlings of herbs and vegetables which are harvested at the development stage of their two cotyledonary leaves, or sometimes at the emergence of their rudimentary first pair of true leaves. They are functional foods, the consumption of which is steadily increasing due to their high nutritional value. The species of the Brassicaceae family are good sources of bioactive compounds, with a favorable nutritional profile. The present study analyzed some phytochemical compounds with nutritional values, such as chlorophylls, polyphenols, carotenoids, anthocyanins, ascorbic acid, total and reducing sugars, and the antioxidant activity of five Brassicaceae species: broccoli (*Brassica oleracea* L.), daikon (*Raphanus raphanistrum* subsp. *sativus* (L.) Domin), mustard (*Brassica juncea* (L.) Czern.), rocket salad (*Eruca vesicaria* (L.) Cav.), and watercress (*Nasturtium officinale* R.Br.). Broccoli had the highest polyphenol, carotenoid and chlorophyll contents, as well as a good antioxidant ability. Mustard was characterized by high ascorbic acid and total sugar contents. By contrast, rocket salad exhibited the lowest antioxidant content and activity. The essential oil (EO) composition of all of these species was determined in order to identify their profile and isothiocyanates content, which are compounds with many reported health benefits. Isothiocyanates were the most abundant group in broccoli (4-pentenyl isothiocyanate), mustard (allyl isothiocyanate), and watercress (benzyl isothiocyanate) EOs, while rocket salad and daikon exhibited higher contents of monoterpene hydrocarbons (myrcene) and oxygenated diterpenes (phytol), respectively. Broccoli microgreens exhibited the overall best nutritional profile, appearing as the most promising species to be consumed as a functional food among those analyzed.

## 1. Introduction

In the new century, the global population needs new sources of food with peculiar nutraceutical properties that can be consumed easily. In this scenario, microgreens could be regarded as one of the main protagonists of this imminent evolution [1].

Microgreens are young shoots of horticultural and herbaceous species; they consist of the fully developed cotyledons and the first true leaves, differing from sprouts since roots are eliminated before their consumption as food [2]. They are typically consumed between 7 and 14 days after germination, when—despite their small size—they already have an intense flavor, a crunchy texture, a very vivid color, and—most importantly—they show interesting beneficial properties for human health, making them an opportunity for both the food industry and the pharmaceutical market [3]. Thanks to their significant content of bioactive compounds, in fact, microgreens can be considered functional foods, representing sources of protein, fat, vitamins, and sugars [4,5]. Compared to the seeds and adult plants of the same species, microgreens hyperaccumulate phytochemicals by, on average, 10 times more. In particular, they contain few antinutrients, and they are rich in amino acids and mineral salts (Ca, Mg, Fe, Mn, Zn, Se, and Mo), as well as secondary metabolites, such as polyphenols, anthocyanins, carotenoids and ascorbic acid in higher concentrations compared to mature plants [2,6]. On the other hand, these shoots contain lower nitrate levels than their mature-leaf counterparts [7]. Researchers have provided a lot of information and reviews on the nutritional traits of microgreens, because they are affected by different cultivars or landraces, plant growth stages [8], and environmental conditions.

All of these compositionally-positive aspects are coupled with a relatively easy production process, as they only need water, light, and a substrate to grow on. Different cultivation systems can affect their production, such as the use of soilless practices (peat moss, vermiculite, and perlite). The important role played by different light intensities (sunlight or UV) and light-emitting diodes in the growth of sprouts and microgreens has recently been reported [9,10]. The possibility of micro-scale production and their high nutritional value make them excellent candidates for the preparation of functional salads for astronauts [11]. Furthermore, their production can also be intended for consumers who need special diets, such as in the case of patients with kidney problems or nutritional deficiencies. Microgreens, for example, can be grown on substrates enriched with iron, and therefore support the resolution of iron-deficiency–related diseases [12], or on substrates almost totally starved of potassium, allowing patients suffering from impaired kidney function to include vegetables in their diet without risks [11]. Microgreens also offer the opportunity to increase the sustainability of vegetable production. Broccoli microgreens, for example, require approximately 200 times less water and need 95% less time to grow than mature broccoli; moreover, they do not require the application of fertilizers or pesticides [12].

The vegetable species used in microgreen production belong to several botanical families, such as Asteraceae, Apiaceae, Amarillydaceae, Amaranthaceae, Brassicaceae, Cucurbitaceae, and Fabaceae, for which several different phytochemicals with antioxidant and healthy properties have been reported. Most of the published studies involved Fabaceae and Brassicaceae [2,13,14]; microgreens belonging to the latter family, in particular, were reported as good sources of K, Ca, Fe and Zn [15]. The phytochemical composition of Brassicaceae varies considerably as a consequence of the plant growth stage and the analyzed species [16]. Glucosinolates are a class of sulfur-rich thioglucoside natural products, present in the Brassicaceae members and related families [17], of which over 120 individual compounds have been identified [18]. They are stored in cell vacuoles; once the plant cell is damaged, they are exposed to myrosinases, a pool of thioglucosydase enzymes produced by thioglucosinolates-synthesizing plants, which release isothiocyanates and other hydrolyzed derivatives [17,19]. These hydrolysis compounds, used as a defense mechanism against herbivores and parasites in glucosinolates-producing species [20], are also important in human health, as they have been reported as cancer-preventing compounds of great nutritional value in a balanced and healthy diet [21]. Their release during the consumption of these species is triggered by mastication, during which the same release of glucosinolates from the vacuoles and their subsequent contact and hydrolysis by myrosinases occur, thus releasing the hydrolysis products. Both glucosinolates and isothiocyanates are involved in the perception of the distinctive aroma and taste of Brassicaceae species [22,23]; in particular, the former are mainly responsible for their bitterness, while the latter for their pungency, although this sometimes varies for some individual compounds of this chemical class [24].

In the present study, microgreens of five Brassicaceae species were analyzed: broccoli (*Brassica oleracea* L.), daikon (*Raphanus raphanistrum* subsp. *sativus* (L.) Domin), mustard (*Brassica juncea* (L.) Czern.), rocket salad (*Eruca vesicaria* (L.) Cav.), and watercress (*Nasturtium officinale* R.Br.). For each species, selected phytochemical compounds with nutritional value (chlorophylls, polyphenols, carotenoids, anthocyanins, ascorbic acid, total and reduced sugars), their antioxidant activity, and the composition of their essential oils were evaluated. The aim was to assess the individual characteristics of each species, in order to obtain a more complete overview of their potential nutritional value, as well as their aroma-distinctive compounds.

## 2. Materials and Methods

### 2.1. Plant Material

The plant material was provided by Azienda Agricola ‘L’Ortofruttifero’ (San Giuliano Terme, Pisa, Italy; GPS N 43.7694938, E 10.3664231,15).

The seeds of broccoli (*Brassica oleracea* var. *italica* L.), daikon (*Raphanus raphanistrum* var. *sativus* (L.) Domin), mustard (*Brassica juncea* var. *mairei* (L.) Czern.), rocket salad (*Eruca vesicaria* var. *sativa* (L.) Cav.), and watercress (*Nasturtium officinale* var. *microphyllum* R.Br.) were sown in May and June 2019 in 10 cm diameter pots, and were left to germinate for two days at 24 °C on a germination plateau. The microgreens were grown in a Brill Orto-pack Bio (MT) (Agrochimica S.p.A., Bolzano, Italy) organic substrate consisting of blonde peat (fraction 0–5), coir (light fraction) and black peat (fraction 0–6), with a bulk density of 270-320 g/L, an air volume of 20/25%, and a water retention capacity of 5.8 g/g. The substrate was characterized as follows: pH 5.5–6.5; EC < 1 mS/cm; N 365 mg/L; P 125 mg/L; K 167.5 mg/L; Mg 12mg/L; Fe 15mg/L; S 38mg/L. No fertilizers or nutrient solutions were provided. Irrigation took place daily by sprinkling without the use of fertilizers or nutritional solutions. On the third day, the pots were moved to the greenhouse at 30 °C during the day and 20 °C at night, with a photoperiod of 16 h:8 h (day:night). The light intensity was between 100.000 and 150.000 lux. All of the species were harvested after 14 days of germination, when they presented fully expanded and turgid cotyledons and the presence of their first true leaves. Harvesting was achieved by cutting the hypocotyls, the length of which ranged between 5 cm in broccoli and 7 cm in watercress. The fresh samples were weighed (Mo) and dried in an oven for 24 h at 60 °C, to their final constant dried weight (M). The percentage of dry matter (DM) was determined according to the following equation: DM (%) = [(Mo − M)/Mo] × 100; the percentage of their water content (WC) was determined as WC % = 100 (%) − DM (%). The collected material was used fresh or stored at −80 °C for further analysis.

**Figure 1:**
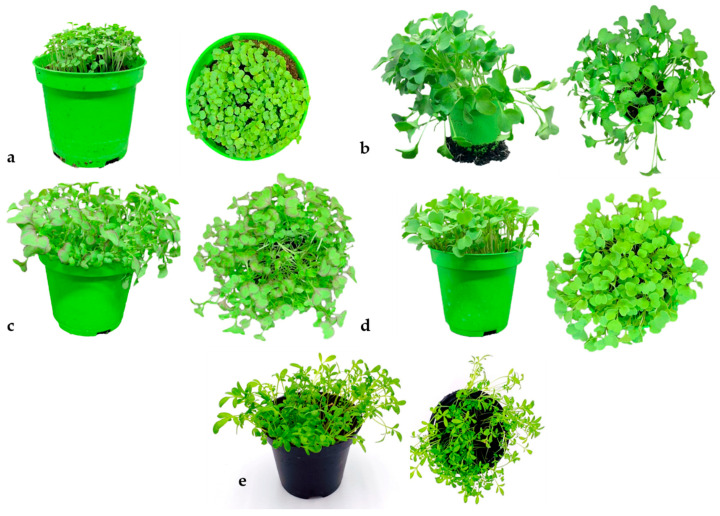
Photos of the analyzed plant material. (**a**) Broccoli (*Brassica oleracea* L.); (**b**) Daikon (*Raphanus raphanistrum* subsp. *sativus* (L.) Domin); (**c**) Mustard (*Brassica juncea* (L.) Czern.); (**d**) Rocket salad (*Eruca vesicaria* (L.) Cav.); (**e**) Watercress (*Nasturtium officinale* R.Br.).

All of the analyzed species are shown in the phenological state they were at when they were analyzed in Figure 1a–e.

### 2.2. Biochemical Analyses

The aerial part of the fresh seedlings (200 mg) was used for the determination of the pigments (chlorophylls and carotenoids) by extraction in 100% methanol, following the known literature [25]. Fresh samples (200 mg) were extracted by homogenization in 2 mL 70% (*v/v*) methanol. After 30 min of incubation at 4 °C, the extracts were centrifuged at the maximum speed for 10 min, and the supernatants were also used for the determination of the total polyphenols by the Folin–Ciocalteau method, and the determination of the 2,2-diphenyl-1-picrylhydrazyl radical (DPPH) antiradical activity, according to the published protocols [26], using an Ultraviolet-Visible (UV-VIS) spectrophotometer (SHIMADZU UV-1800, Shimadzu Corp., Kyoto, Japan). The DPPH activity was determined as IC_50_ (mg/mL), namely the extract concentration required to obtain 50% of antioxidant activity. The extracts were also tested with a Ferric ion Reducing Antioxidant Power (FRAP) antioxidant assay in order to confirm the antioxidant activity [27]. The total polyphenols content (TPC) was expressed as mg gallic acid equivalents (GAE) per g of fresh weight (FW). The anthocyanins were extracted from 0.1 g fresh leaves in ethanol/HCl (99/1, *v/v*), and were used to read the absorbance at 535 nm [28]. The total anthocyanin content (TAnth) was expressed as μg malvin-chloride equivalents (ME) per g of FW. The data presented are the mean of three independent replicates.

The reduced and total ascorbic acid (AsA and AsA_TOT_) were quantified according to the method of Kampfenkel et al. [29]. Fresh samples (200 mg) were extracted in 2 mL 6% (*w/v*) trichloroacetic acid (TCA) solution, as described by Degl’Innocenti et al. [30]. The data are reported as μg of AsA_TOT_ per g of FW.

### 2.3. Reducing and Total Soluble Sugars Quantification

The reducing sugars were determined using 3,5-dinitrosalicylic acid (DNS) reagent, prepared as described by Teixeira et al. [31]: 10 g/L DNS, 16 g/L sodium hydroxide, and 300 g/L sodium potassium tartrate. The fresh samples were extracted according to Li et al. [32], with some modification. In brief, 200 mg were extracted at 80 °C for 30 min with 2 mL 80% ethanol and 1 mL hot distilled water. After 10 minutes at the maximum centrifuge speed, the supernatant was collected, and the extraction procedure was repeated once. The final extraction volume was taken up to a volume of 10 mL with distilled water, and was used to determine both the reducing and total soluble sugars.

The reducing sugar determinations were performed according to Teixeira et al. [31] as follows: 0.4 mL DNS solution was added to 0.2 mL sample, then boiled at 100 °C for 5 min. The samples were cooled to room temperature in ice, and 1 mL distilled water was added. The absorbance was measured at 540 nm.

The total soluble sugars were spectrophotometrically estimated as reported in Das et al. [33], with some modifications. In brief, 0.8 mL 0.2% (*w/v*) anthrone solution were added to 0.2 mL sample, and—after 12 min of incubation at 90 °C—the absorbance was read at 620 nm.

For both analyses, three biological replicas were used, and the data were expressed as mg glucose per g FW.

### 2.4. Essential Oil Hydrodistillation and Analysis by Gas-Chromatography-Mass Spectrometry (GC-MS)

For each species, 300 g fresh leaves were cut and subjected to hydrodistillation in a Clevenger-type apparatus for 3 hours. As all of the samples did not yield enough essential oil to be gathered in its purity, 0.5 mL of *n*-hexane HPLC grade was inserted into the Clevenger apparatus in order to capture the volatile fraction hydrodistilled from the plant material. Thus, the hydrodistillation yield could not be calculated (<0.1% *w*/*w*). All of the hydrodistillations were performed in triplicates.

Gas-Chromatography—Electron-Impact Mass Spectrometry (GC/EI-MS) analyses were performed with an Agilent 7890B gas chromatograph (Agilent Technologies Inc., Santa Clara, CA, USA) equipped with an Agilent HP-5MS (Agilent Technologies Inc., Santa Clara, CA, USA) capillary column (30 m × 0.25 mm; coating thickness 0.25 μm) and an Agilent 5977B single quadrupole mass detector (Agilent Technologies Inc., Santa Clara, CA, USA). The operating conditions were as follows: oven temperature programmed rising from 60 °C to 240 °C at 3 °C /min; injector temperature 220 °C; transfer-line temperature 240 °C; carrier gas He (1 mL/min). For each EO, the injection volume was 1 μL.

The identification of the constituents was based on the comparison of their retention times with those of the authentic samples (when available), comparing their linear retention indices to the series of C9–C25 *n*-hydrocarbons. Computer matching was also used against a commercial (NIST 14) and a laboratory-developed mass spectra library, which was built up from pure substances and the components of commercial essential oils of known composition, and MS literature data [34,35].

### 2.5. Statistical Analysis

The biochemical data were statistically analyzed by one-way analysis of variance (ANOVA) with software Past3, version 3.15., using either Tukey Honestly Significant Difference (HSD) or the Mann-Whitney test according to the variance homogeneity (Levene test), with a cut-off significance of *p* < 0.05 (letters). The linear correlation between the antioxidant constituents and antioxidant scavenging activity (DPPH) was determined using Microsoft Excel ^®^ 2013 (Microsoft Corporation, Redmond, WA, USA).

The hierarchical cluster (HCA) and principal component (PCA) analyses of the complete EO compositions were performed in JMP 13.2.0 (SAS Institute, Cary, NC, USA). The HCA was conducted using Ward’s algorithm on normalized data, using Euclidean distances as a measure of similarity. To perform the PCA, linear regressions were operated on the mean-centered data of the covariance matrix, in order to select the two highest principal components (PCs). The collected data-set was a 5 × 50 matrix (5 samples, 50 individual compounds). This unsupervised method reduced the dimensionality of the multivariate data of the matrix while preserving most of the variance [36]. The chosen PC1 and PC2 covered 44.8% and 31.8% of the variance, respectively, for a total of 76.6% of the observed data.

## 3. Results

### 3.1. Biochemical Analyses

The water content, the dry matter percentage of fresh samples, their biochemical metabolites (reducing and total sugars, chlorophylls a and b, total chlorophylls, Chl a/ Chl b ratio, total carotenoids, total polyphenols, total anthocyanins, reduced and total ascorbic acid, and their relative ratio), and their antioxidant activity (DPPH and FRAP assays) are reported in Table 1.

The highest water content (%) was found in white radish (93.59%), followed by rocket salad (93.31%), broccoli (91.06%), watercress (88.94%), and finally mustard (86.75%). In correlation with the water content (inversely proportional), the percentage of the dry weight was quite high in the case of mustard and watercress, followed by broccoli; the lowest values were measured for rocket and daikon.

The highest reducing sugar content (mainly due to glucose and fructose) was detected in watercress and rocket salad (8.44 and 7.98 mg GLU/g FW, respectively), while daikon and broccoli contained an almost halved content (4.47 and 4.66 mg GLU/g FW respectively) of these compounds. Considering the total sugar content, mustard showed the highest content by a significant amount (58.11 mg GLU/g FW), threefold higher than that of broccoli, rocket salad, and watercress, and twofold higher than that of daikon.

The growth condition of the microgreens can be evaluated by their chlorophyll content. The highest (statistically different) content of chlorophyll a (Chl a) was found in mustard (982.3 µg/g FW), followed by broccoli (737.8 µg/g FW), and rocket salad, daikon, and watercress, which showed similar values to one another (681.8, 623.6 and 584.8 µg/g FW, respectively). A similar trend was observed for the content of chlorophyll b (Chl b), of which mustard showed the highest level (409.2 µg/g FW), followed by watercress (233.0 µg/g FW) and broccoli (223.9 µg/g FW), while its lowest concentration was found in daikon and rocket salad (170.3 and 131.8 µg/g FW, respectively). The total chlorophyll content reflects the sum of the single chlorophyll content, of which mustard showed the highest content, while daikon showed the lowest. The Chl a/Chl b ratio was in the range of 2.51–3.66, with the exception of rocket salad (5.22); the generally accepted ratio which is considered an index of optimal plant growth is above 2.5–3.

The total carotenoids amount was significantly lower in watercress (96.9 µg/g FW), while the other Brassicaceae microgreens showed similar values, in the range of 175–217 µg/g FW.

In the evaluation of their nutritional value, antioxidant compounds such as polyphenols, anthocyanins, AsA, and carotenoids play an important role, and are associated with the antioxidant activity. The highest total polyphenol content (TPC) was detected in broccoli (3.63 µg/g FW), followed by daikon, watercress, and rocket salad. Mustard showed the lowest amount (1.02 µg/g FW), which was statistically different from the other microgreens.

The highest level of anthocyanins was found in mustard (405.52 µg/g), followed by broccoli (172.51 µg/g FW). The lowest levels, on the other hand, were detected in daikon and watercress (57.56 and 52.28 µg/g FW, respectively). Rocket salad (42.26 µg/g FW) showed the lowest anthocyanin concentration, which was statistically different from the other species.

The largest vitamin C (total AsA) content was detected in mustard (606.87 µg/g FW). In broccoli, daikon, and watercress, the AsA content was about fourfold lower than that in mustard, but very similar to each other (124.1–137.52 µg/g FW). A rather low content was detected in rocket salad (29.67 µg/g FW). Lastly, the reduced AsA content was extremely high in mustard (366.07 µg/g FW), and about 10 times lower in rocket salad (25.86 µg/g FW) and watercress (38.55 µg/g FW).

The highest antioxidant activity was observed in daikon, broccoli and watercress, in both the DPPH and FRAP assays, while rocket salad and mustard showed significantly lower activities.

### 3.2. Essential Oil Compositions

The complete essential oil (EO) compositions are reported in Table 2. Overall, 50 compounds were detected: 13 in broccoli, 30 in daikon, 17 in mustard, 9 in rocket salad, and 9 in watercress. The percentages of the identified compounds varied between 96.4% (daikon) and 100.0% (watercress).

Broccoli’s EO was constituted of isothiocyanates for over 97% of its total, with 4-pentenyl, phenethyl, and 3-butenyl isothiocyanates collectively representing over 95% of the total composition. Among the non-isothiocyanates compounds, only 5-cyano-1-pentene exhibited a relative abundance over 1.0%.

Daikon’s EO exhibited the most varied composition, both in terms of individual compounds and chemical classes. Oxygenated diterpenes, of which phytol was the only representative, were detected as the most abundant chemical group (29.0%). Non-terpene derivatives closely followed (27.7%), with (*Z*)-3-hexen-1-ol (11.0%) and nonanal (5.3%) as the most abundant.

Isothiocyanates represented over 40% of mustard’s EO composition; among them, allyl and 3-butenyl isothiocyanates were the most abundant, accounting for up to 22.7% and 14.1%, respectively. Phytol, however, showed the highest relative abundance in this EO as an individual compound, as it was detected with a relative abundance of 28.4%. Non-terpene derivatives added up to 12.7% of the total composition, with (*Z*)-3-hexen-1-ol (7.2%) and its acetic ester (2.4%) as the most abundant. Among the monoterpene hydrocarbons, only limonene was detected, with a relative concentration of 3.6%.

Rocket salad’s EO was dominated by monoterpene hydrocarbons, mainly represented by myrcene (83.7%) and limonene (7.5%). The sesquiterpene hydrocarbons group followed, and was only represented by β-caryophyllene (4.4%) and α-humulene (1.3%).

Over 65% of watercress’ EO was constituted of isothiocyanates, among which benzyl isothiocyanate was the only detected compound of this chemical class. Benzylnitrile followed as the second most abundant compound in the EO, accounting for up to 26.0%. Phytol, the sole oxygenated diterpene detected in this EO, showed a relative concentration of 3.3%.

**Figure 2:**
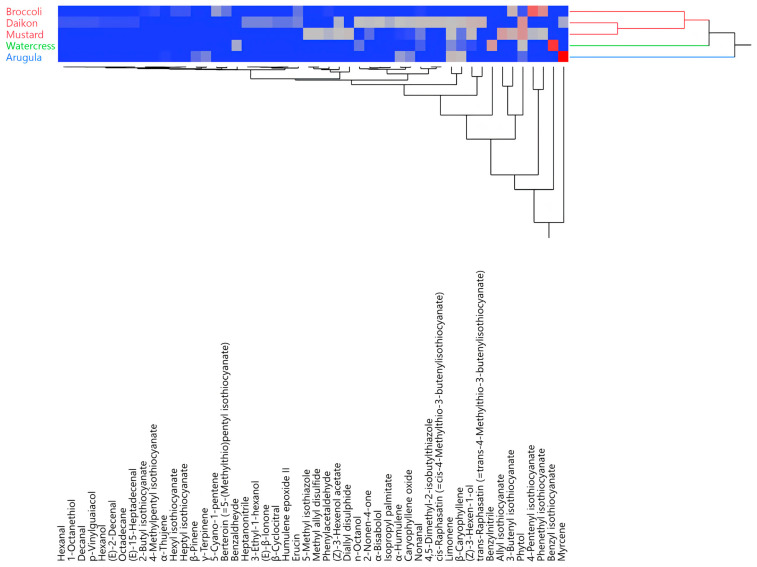
Two-way dendrogram obtained using the hierarchical cluster analysis performed on the complete essential oil (EO) compositions for all of the analyzed species.

The hierarchical cluster analysis (HCA) of all of the EO compositions distributed the samples into two main macro-clusters (Figure 2). The first macro-cluster comprises two sub-groups (red and green), while the second comprises only one (blue). Rocket salad’s EO was clustered by itself in the blue group. Watercress’ EO represented a group of its own, as well, but its composition was closer to that of the red samples, as they were all in the same macro-cluster.

**Figure 3:**
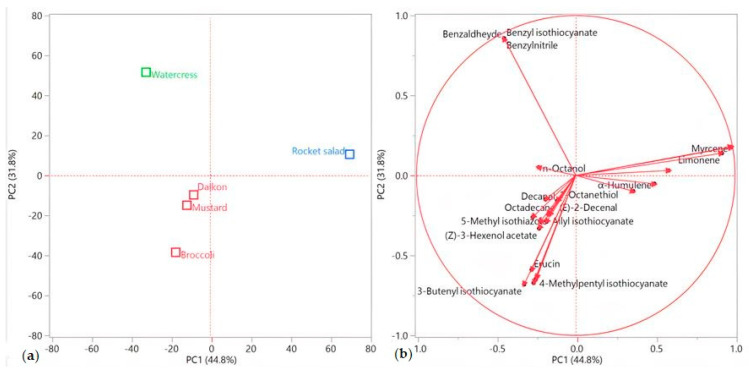
Score (**a**) and loadings (**b**) plots obtained using the principal component analysis performed on the complete EO compositions for all of the analyzed species.

This pattern was confirmed by the principal component analysis (PCA), as shown in Figure 3a,b. Rocket salad was, indeed, plotted by itself in the upper right (PC1 and PC2 > 0) of the score plot (Figure 3a), due to its extremely relevant presence of myrcene and limonene (Figure 3b). Watercress’ EO was the only sample plotted in the upper left (PC1 < 0, PC2 > 0) quadrant (Figure 3a), where its positioning was chiefly determined by the vectors of its two major compounds (benzyl isothiocyanate and benzylnitrile, Figure 3b). All of the other samples were grouped in the lower left (PC1 and PC2 < 0) quadrant (Figure 3a), towards which basically all of the other compound vectors pointed (Figure 3b).

## 4. Discussion

Plant water content is an important factor in establishing the turgor of plant cells; an adequate presence of water in plant cells ensures optimal metabolism, in terms of (i) the absorption of salts from the outside (soil or substrate), (ii) proper evapotranspiration, (iii) the regulation of the leaf temperature, and iv) the photosynthetic process [37]. The results obtained in the present work evidenced that the water content of all of the microgreens was quite high, although they were grown in soil instead of the more commonly used hydroponic or soilless technique [5,11,38]. The highest percentage of water was measured in daikon microgreens (93.59%), as was predictable due to their fleshy leaves, while in the other analyzed species it was higher than 85%. The percentage of dry matter was low and stable in all of the species, and it was comparable with that of some already-published studies on microgreens, especially for daikon, broccoli [6,15], and rocket salad [6]. However, watercress and mustard showed a higher water content compared to some other reports [15,39].

Color is a fundamental characteristic which is linked to the quality of vegetables, and which strongly affects consumers’ preferences. In the case of microgreens, the color of the stem, cotyledons and/or of the first true leaves is of great importance; some producers offer heterogeneous chromatic products, and they recall it with fancy names [40]. Chlorophylls and carotenoids are recognized as the major pigments affecting the visual (color) appearance of green sprouts. The total chlorophyll content (the sum of Chl a and b) was 793 µg/g for daikon, and 1391 µg/g was measured in mustard; these results are in agreement with those of Kopsell et al. [41]. The total carotenoid content of the examined microgreens did not differ among the species, with the exception of watercress. However, in the present report, some data differed from other data that have already been published; these discrepancies can be attributed to different cultivation techniques—e.g., greenhouse conditions—and to differences in the used soil. In the present work, the microgreens were cultivated in an organic substrate instead of the frequently used soilless or hydroponic culture [38]. Moreover, the harvest time (the day of cultivation of the microgreens at harvest) is not reported frequently enough to allow a comparison [5,6,38,39]. Anthocyanins were detected in a high concentration in mustard; their leaf margins, indeed, showed a red color, which is retained in the adult phase [42]. Broccoli followed, and while daikon, rocket salad, and watercress showed low contents, as was already demonstrated in similar growth conditions [43].

The results obtained in the present study showed that the different microgreen species provided extremely varying amounts of vitamin C. Mustard was characterized by higher levels of total ascorbic acid (AsA) and of its reduced form compared to the other species under evaluation, which is in agreement with other reports [43].

Carotenoids, anthocyanins, polyphenols, and AsA are known as antioxidant molecules, and the antioxidant activity is an important feature for the use of microgreens as functional foods. In this work, the antioxidant activity was detected by FRAP assay and the IC_50_ of DPPH radical scavenging. The obtained results are in agreement with those of other reports [44]; as such, in this discussion, we highlighted the correlation between the antioxidant activity and the correlated compounds. The details of the correlation analysis are provided in Table S1. Daikon microgreens exhibited the highest antioxidant activity, which was essentially due to their high level of polyphenols (comparable to that of broccoli), ascorbic acid, and carotenoids (similar to that of rocket salad and broccoli). A strong correlation (Table S1) was confirmed for polyphenols and ascorbic acid. On the other hand, for adult daikon plants, the antioxidant activity is attributable to their high level of both polyphenols and anthocyanins [45]. A high antioxidant activity was also highlighted in broccoli microgreens, which was only slightly lower than that of daikon, as reported by Paradiso et al. [46] and Di Bella et al. [47]. This activity can be attributed to polyphenols, carotenoids, and anthocyanins, and it reflects the results obtained by Jagdish et al. [48]. However, during growth, the adult plants showed high levels of polyphenols and anthocyanins, but not of carotenoids [49]. Watercress’ radical-scavenging activity was mainly attributed to its consistent level of carotenoids, which is in agreement with Amiri [50]. On the other hand, the adult watercress plant has a low content of carotenoids, while the content of anthocyanins and polyphenols is high [51]. The low concentration of polyphenols and carotenoids can play a role in the very low scavenging activity detected in mustard [6], although it showed the highest content of ascorbic acid and anthocyanins compared to the others. The adult mustard plant maintains elevated levels of ascorbic acid, and its carotenoid content increases [52]. A very low scavenging activity was also detected in rocket salad, due to its low level of both anthocyanins and ascorbic acid, as confirmed by previous studies carried out for the evaluation of its antioxidant activity [53]. It also exhibited high levels of carotenoids, which were comparable to those of broccoli and daikon plants. The adult plant, like the microgreen, according to Abdalla [54], has low levels of ascorbic acid.

Reports on the EO compositions for microgreens are lacking; as such, to the best of our knowledge, this is the first study reporting their composition for all of the analyzed species. For this reason, our compositional comparison with already-published results could only be performed with adult plants, when such information was available.

For broccoli microgreens, isothiocyanates dominated their EO composition; these compounds were also detected with relevant relative concentrations in adult specimens from France and Italy, although their compositions were, instead, dominated by cyanides [55]. The most abundant compound in broccoli microgreens’ EO which was measured present study was 4-Pentenyl isothiocyanate, which is reported to be an aroma-active compound, the contribution of which is defined as acrid, pungent, and mustard-like, and it is also found in wasabi, in which it contributes to its pungency [24]. Phenethyl isothiocyanate follows as the second most represented compound; its aroma contribution is also reported as pungent, and it is also described as being able to cause a tingling sensation in the mouth [24]. Interestingly, for *B. oleracea* var. *italica*, glucoraphanin, and other isothiocyanates showed a decrement from the start of seed germination to the flowering stages [56]; isothiocyanates might, thus, be at their peak concentration in the microgreen’s developmental stage.

For daikon, as far as we are aware, no published studies are available on its EO composition. Phytol was the most abundant compound in daikon microgreens EO; this oxygenated diterpene is described as having a delicately balsamic and floral aroma [57]. *trans*-Raphasatin followed, and its aroma contribution is reported to be pungent [24]. (*Z*)-3-Hexen-1-ol was the third most abundant compound in this EO; its odor strength is high, and it is characterized as green and pungent [58].

For mustard, the microgreens’ EO composition was mainly rich in phytol, closely followed by allyl isothiocyanate. The latter is characterized by a bitter, pungent, and sulfur-like aroma, very similar to that of the third most abundant compound, 3-butenyl isothiocyanate, which is described as wasabi-like [24]. The significant presence of allyl isothiocyanate in mustard’s EO is in accordance with the published studies on adult mustard plants from China [59] and Korea [60].

The absence of isothiocyanates in the rocket salad microgreens’ EO was quite surprising. Instead, the EO analysis revealed a composition dominated by monoterpene hydrocarbons. Myrcene, a monoterpene hydrocarbon with an herbaceous and sweetly balsamic aroma contribution [61], accounted for over 83% of these microgreens’ EO. For adult rocket salad plants, instead, the reported EO compositions are mainly composed of isothiocyanates, especially 4-methylthiobutyl isothiocyanate, and nitriles [62,63]. This noteworthy compositional difference in EO compositions depending on the growth stage of plants of this species might be due to a later development of the isothiocyanates’ biosynthesizing enzymes. For *E. sativa*, indeed, as reported by Falk et al. (2004), the first phase of the isothiocyanates biosynthesis, namely the amino acids’ side-chain elongation, takes place in two separate steps, each controlled by a different pool of enzymes [17]. Thus, the absence of isothiocyanates in the rocket salad microgreens’ EO of the present study might be due to the lack of one of the two pools of enzymes, which we hypothesized could be developed in a later growth stage.

Watercress microgreens’ EO was mainly rich in isothiocyanates and nitriles. Among the former, the most abundant was benzyl isothiocyanate, the aroma of which is described as pungent [24]; benzylnitrile was the only detected compound among the latter. The studies published in the literature about Iranian adult specimens EO are conflicting: one study found phytol as the most abundant compound [64], while another sample was mainly rich in myristicin [50].

## 5. Conclusions

In the last decade, the interest in microgreens has grown, as they meet consumers’ preference for their novelty, palatability, and ease of use, all coupled with desirable health-related benefits ensured by their nutritionally valuable compositions. They are also interesting for the producers, as they have very few production requirements, and they reach their ideal growth for consumption quite quickly. Many species are eligible for microgreens production; however, Brassicaceae spp. are among the most popular.

All of the five Brassicaceae species analyzed in the present work showed noteworthy nutritional characteristics, and they all had quite comparable antioxidant properties, which were due to the different combinations of antioxidant compounds present in their compositions. Thus, the choice between these five species could be mainly driven by consumers’ preference of their aroma profile. Broccoli and watercress were characterized by strong and pungent aroma-active compounds, as are typical of the expected Brassicaceae bouquet. Mustard microgreens, while still rich in allyl isothiocyanate’s pungent flavor, had a more delicate aroma, given the relevant presence of phytol; the same intermediate character was found for daikon microgreens. Rocket salad microgreens, instead, were characterized by the absence of isothiocyanates, as is typically associated to Brassicaceae’s peculiar aroma.

However, strictly taking into account the nutritional aspect of the analyzed samples, broccoli (*B. oleracea* L.) microgreens showed the strongest antioxidant power, as well as the largest isothiocyanates content, thus coupling the benefits of the radical-scavenging properties of its polyphenols and the cancer-preventing ability of its isothiocyanates.

## Figures and Tables

**Table 1:** Biochemical data for all of the evaluated parameters in the analyzed Brassicaceae species.

Parameters	*B. oleracea*	*R. raphanistrum*	*B. juncea*	*E. vesicaria*	*N. officinale*
	Broccoli	Daikon	Mustard	Rocket Salad	Watercress
Water content (%)	91.06	93.59	86.75	93.31	88.94
Dry matter (%)	8.94	6.41	13.25	6.69	11.06
Reducing sugars (mg GLU/g FW)	4.66 ± 0.24 ^b^	4.47 ± 0.21 ^b^	6.40 ± 0.59 ^ab^	7.98 ± 0.35 ^a^	8.44 ± 0.95 ^a^
Total sugars (mg GLU/g FW)	16.48 ± 1.26 ^ca^	28.27 ± 1.30 ^b^	58.11 ± 4.19 ^a^	16.99 ± 0.75 ^c^	18.87 ± 1.26 ^bc^
Chl a (μg/g FW)	737.83 ± 32.84 ^b^	623.55 ± 30.69 ^bc^	982.29 ± 24.51 ^a^	681.83 ± 21.04 ^bc^	584.76 ± 14.06 ^c^
Chl b (μg/g FW)	223.92 ± 20.14 ^b^	170.29 ± 7.14 ^c^	409.15 ± 29.78 ^a^	131.81 ± 6.66 ^d^	233.04 ± 5.57 ^b^
Chl_TOT_ (μg/g FW)	961.75 ± 45.45 ^b^	793.83 ± 26.48 ^c^	1391.44 ± 41.15 ^a^	813.65 ± 23.50 ^bc^	817.80 ± 18.18 ^bc^
Chl a/Chl b	3.37 ± 0.22b ^b^	3.66 ± 0.19 ^b^	2.51 ± 0.18 ^c^	5.22 ± 0.27 ^a^	2.51 ± 0.05 ^c^
TCar (μg/g FW)	217.30 ± 12.00 ^a^	190.58 ± 8.21 ^a^	175.04 ± 15.83 ^a^	213.30 ± 5.90 ^a^	96.87 ± 6.29 ^b^
TCar/Chl_TOT_	0.23 ± 0.01 ^b^	0.24 ± 0.01 ^ab^	0.12 ± 0.01 ^c^	0.26 ± 0.00 ^a^	0.12 ± 0.01 ^c^
TPC (mg GAE/g FW)	3.63 ± 0.11 ^a^	3.25 ± 0.12 ^ab^	1.02 ± 0.05 ^c^	2.98 ± 0.11 ^b^	3.08 ± 0.27 ^b^
TAnth (μg ME/g FW)	172.51 ± 24.37 ^b^	57.56 ± 4.52 ^cd^	405.52 ± 31.55 ^a^	42.26 ± 7.71 ^d^	52.28 ± 4.32 ^cd^
Reduced ascorbic acid (μg AsA/g FW)	98.27 ± 10.7 ^b^	93.9 ± 5.12 ^b^	366.07 ± 100 ^a^	25.86 ± 3.12 ^d^	38.55 ± 3.66 ^c^
Total ascorbic acid (μg AsA_TOT_/g FW)	124.1 ± 10.77 ^b^	125.58 ± 8.15 ^b^	606.87 ± 71.89 ^a^	29.67 ± 3.86 ^c^	137.52 ± 14.59 ^b^
AsA/AsA_TOT_	0.79 ± 0.03 ^a^	0.76 ± 0.09 ^ab^	0.57 ± 0.09 ^b^	0.87 ± 0.10 ^a^	0.29 ± 0.04 ^c^
DPPH radical scavenging assay (IC_50_ mg/mL)	3.93 ± 0.27 ^b^	2.93 ± 0.33 ^b^	10.54 ± 1.00 ^a^	12.11 ± 0.50 ^a^	4.26 ± 0.42 ^b^
Antioxidant activity—FRAP assay (mmol Fe2/g FW)	8.6 ± 0.3 ^a^	7.6 ± 0.1 ^a^	3.2 ± 0.2 ^b^	2.1 ± 0.1 ^b^	8.6 ± 0.3 ^a^

Chl, chlorophyll; TCar, total carotenoids; TPC, total polyphenols content; TAnth, total anthocyanins; AsA, ascorbic acid; GLU, glucose; GAE, gallic acid equivalent; ME, malvin-chloride equivalent; DPPH, 2,2-diphenyl-1-picrylhydrazyl radical; FRAP, Ferric Reducing Antioxidant Power; different superscript letters indicate a significant difference at *p* < 0.05.

**Table 2:** Complete EO compositions for all of the analyzed species.

Compounds	l.r.i. ^a^	Relative Abundance (%) ± SD				
		Broccoli	Daikon	Mustard	Rocket Salad	Watercress
Hexanal *	802	- ^b^	0.3 ± 0.46	-	-	-
5-Cyano-1-pentene	853	1.3 ± 0.03	-	-	-	-
(*Z*)-3-Hexen-1-ol *	857	-	11.0 ± 1.37	7.2 ± 0.25	-	0.2 ± 0.3
5-Methyl isothiazole	862	-	-	3.1 ± 0.75	-	-
Hexanol *	871	-	0.2 ± 0.32	-	-	-
Allyl isothiocyanate *	892	-	-	22.7 ± 0.41	-	-
Methyl allyl disulfide	920	-	-	2.9 ± 0.22	-	-
2-Butyl isothiocyanate	931	0.1 ± 0.01	-	-	-	-
α-Thujene	933	-	-	-	0.1 ± 0.15	-
Benzaldehyde *	965	-	-	-	-	1.5 ± 0.03
3-Butenyl isothiocyanate	980	11.7 ± 0.02	-	14.1 ± 1.00	-	-
β-Pinene *	981	-	-	-	0.6 ± 0.05	-
Heptanonitrile	985	-	0.9 ± 0.18	-	-	-
Myrcene *	993	-	1.5 ± 1.56	-	83.7 ± 0.51	-
(*Z*)-3-Hexenol acetate	1007	-	1.7 ± 1.05	2.4 ± 0.26	-	-
3-Ethyl-1-hexanol	1031	-	0.9 ± 0.08	-	-	-
Limonene *	1032	-	2.6 ± 0.03	3.6 ± 0.50	7.5 ± 0.03	1.0 ± 0.00
Phenylacetaldehyde *	1047	0.1 ± 0.08	-	1.7 ± 0.07	-	-
γ-Terpinene *	1062	-	-	-	0.9 ± 0.03	-
1-Octanol *	1071	-	2.9 ± 0.00	-	-	0.7 ± 0.04
Diallyl disulphide *	1083	-	-	5.6 ± 1.24	-	-
4-Pentenyl isothiocyanate	1086	50.2 ± 1.22	-	1.8 ± 0.45	-	-
Nonanal *	1104	0.1 ± 0.00	5.3 ± 0.16	0.8 ± 0.13	-	0.4 ± 0.01
2-Nonen-4-one	1128	-	1.9 ± 0.35	0.6 ± 0.16	-	-
1-Octanethiol *	1132	-	0.3 ± 0.44	-	-	-
Benzylnitrile	1140	-	-	-	-	26.0 ± 0.04
4-Methylpentyl isothiocyanate	1166	0.2 ± 0.03	-	-	-	-
Hexyl isothiocyanate *	1199	0.3 ± 0.01	-	-	-	-
Decanal *	1206	-	0.3 ± 0.37	-	-	-
4,5-Dimethyl-2-isobutylthiazole	1220	-	4.4 ± 0.31	-	-	-
β-Cyclocitral *	1222	-	0.7 ± 0.28	-	-	-
(*E*)-2-Decenal	1263	-	0.2 ± 0.22	-	-	-
Heptyl isothiocyanate	1265	0.3 ± 0.01		-	-	-
*p*-Vinylguaiacol	1313	-	0.3 ± 0.36	-	-	-
Benzyl isothiocyanate *	1363	-		-	-	66.4 ± 0.11
*cis*-Raphasatin	1419	-	4.4 ± 0.12	-	-	-
β-Caryophyllene *	1430	-	5.5 ± 0.01	0.7 ± 0.4	4.4 ± 0.43	0.5 ± 0.00
Erucin	1431	0.7 ± 0.01	0.9 ± 0.05	-	-	-
*trans*-Raphasatin	1440	-	12.2 ± 0.97	-	-	-
α-Humulene *	1456	-	2.0 ± 0.08	0.1 ± 0.19	1.3 ± 0.03	-
Phenethyl isothiocyanate *	1465	33.2 ± 0.94	-	3.4 ± 0.76	-	-
(*E*)-β-Ionone	1486	-	0.9 ± 0.11	-	-	-
Berteroin	1554	0.7 ± 0.08	-	-	-	-
Caryophyllene oxide *	1582	-	1.9 ± 0.16	0.2 ± 0.23	1.0 ± 0.04	-
Humulene epoxide II	1608	-	0.7 ± 0.17	-	-	-
α-Bisabolol *	1684	-	2.2 ± 0.13	-	-	-
Octadecane *	1800	-	0.2 ± 0.27	-	-	-
Isopropyl palmitate *	2023	-	1.6 ± 0.44	-	-	-
(*E*)-15-Heptadecenal	2083	-	0.2 ± 0.25	-	-	-
Phytol *	2115	0.3 ± 0.00	29.0 ± 2.47	28.4 ± 4.90	0.5 ± 0.02	3.3 ± 0.22
Monoterpene hydrocarbons		-	2.8 ± 0.30	3.6 ± 0.50	92.8 ± 0.41	1.0 ± 0.00
Sesquiterpene hydrocarbons		-	7.5 ± 0.08	0.9 ± 0.59	5.7 ± 0.40	0.5 ± 0.00
Oxygenated sesquiterpenes		-	4.7 ± 0.45	0.2 ± 0.23	1.0 ± 0.04	-
Oxygenated diterpenes		0.3 ± 0.00	29.0 ± 2.47	28.4 ± 4.90	0.5 ± 0.02	3.3 ± 0.22
Apocarotenes		-	1.6 ± 0.18	-	-	-
Isothiocyanates		97.4 ± 0.12	17.5 ± 1.14	42.0 ± 2.62	-	66.4 ± 0.11
Thiazole derivatives		-	4.4 ± 0.31	3.1 ± 0.75	-	-
Other nitrogen compounds		1.3 ± 0.03	0.9 ± 0.18	-	-	26.0 ± 0.04
Other sulphur compounds		-	0.3 ± 0.44	8.5 ± 1.46	-	-
Other non-terpene derivatives		0.2 ± 0.08	27.7 ± 0.29	12.7 ± 0.38	-	2.9 ± 0.29
Total identified (%)		99.1 ± 0.07	96.4 ± 1.30	99.3 ± 0.14	100.0 ± 0.01	100.0 ± 0.00

^a^ Linear retention index on a HP-5MS capillary column; ^b^ not detected; * compounds for which a pure analytical standard was available for injection.

## Data Availability

Data is contained within the article.

## Supplementary Materials

The following are available online at https://www.mdpi.com/2304-8158/10/2/427/s1, Table S1: Correlation between antioxidants content (total polyphenols and ascorbic acid content) in leaves of five Brassicaceae species (*B. oleracea*, *R. raphanistrum*, *B. juncea*, *E. vesicaria*, *N. officinale*) microgreens and their radical scavenger activity (DPPH assay, IC50).
