# Supplementary material for: Small Functional Foods: Comparative Phytochemical and Nutritional Analyses of Five Microgreens of the Brassicaceae Family
Source: Foods. 2021 Feb 15;10(2):427. doi: 10.3390/foods10020427 (PMC7919663; doi:10.3390/foods10020427)
Supplement: Supplementary file 1 [file foods-10-00427-s001.pdf]

Table S1. Correlation between antioxidants content (total polyphenols and ascorbic acid content) in leaves of five Brassicaceae species (*B. oleracea, R. raphanistrum, B. juncea, E. vesicaria, N. officinale*) microgreens and their radical scavenger activity (DPPH assay, IC_{50}).

**Species**	**Antioxidant**	**Equation**	R^2
B. oleracea—Broccoli	Total polyphenols	y = -1.7568x + 10.341	0.6834
	Total ascorbic acid	y = -34.611x + 7.9447	0.9582
	Total anthocyanins	y = -0.0107x + 5.7968	0.8988
	Total carotenoids	y = -0.028x + 6.4277	0.9850
R. raphanistrum—Daikon	Total polyphenols	y = -2.6326x + 11.496	0.9803
	Total ascorbic acid	y = -70.15x + 9.6086	0.7728
	Total anthocyanins	y = -0.0529x + 6.1427	0.8057
	Total carotenoids	y = -0.0363x + 9.9407	0.5326
B. juncea—Mustard	Total polyphenols	y = -7.715x + 18.755	0.7582
	Total ascorbic acid	y = -5.9945x + 14.504	0.8981
	Total anthocyanins	y = -0.0062x + 13.742	0.7273
	Total carotenoids	y = -0.1688x + 14.125	0.7743
E. vesicaria—Rocket salad	Total polyphenols	y = -4.0734x + 24.233	0.8728
	Total ascorbic acid	y = -135.81x + 15.624	0.7324
	Total anthocyanins	y = -0.0551x + 14.035	0.7427
	Total carotenoids	y = -0.0808x + 29.344	0.9305
N. officinale—Watercress	Total polyphenols	y = -1.5374x + 9.284	0.7404
	Total ascorbic acid	y = -24.142x + 7.5931	0.9422
	Total anthocyanins	y = -0.0466x + 6.3114	0.8315
	Total carotenoids	y = -0.0737x + 11.359	0.8633
